# Toward prediction of abscopal effect in radioimmunotherapy: Pre-clinical investigation

**DOI:** 10.1371/journal.pone.0255923

**Published:** 2021-08-24

**Authors:** Ivaylo B. Mihaylov, Tulasigeri M. Totiger, Teresa M. Giret, Dazhi Wang, Benjamin Spieler, Scott Welford

**Affiliations:** Department of Radiation Oncology, University of Miami Miler School of Medicine, Miami, FL, United States of America; Northwestern University Feinberg School of Medicine, UNITED STATES

## Abstract

**Purpose:**

Immunotherapy (IT) and radiotherapy (RT) can act synergistically, enhancing antitumor response beyond what either treatment can achieve separately. Anecdotal reports suggest that these results are in part due to the induction of an abscopal effect on non-irradiated lesions. Systematic data on incidence of the abscopal effect are scarce, while the existence and the identification of predictive signatures or this phenomenon are lacking. The purpose of this pre-clinical investigational work is to shed more light on the subject by identifying several imaging features and blood counts, which can be utilized to build a predictive binary logistic model.

**Materials and methods:**

This proof-of-principle study was performed on Lewis Lung Carcinoma in a syngeneic, subcutaneous murine model. Nineteen mice were used: four as control and the rest were subjected to combined RT plus IT regimen. Tumors were implanted on both flanks and after reaching volume of ~200 mm^3^ the animals were CT and MRI imaged and blood was collected. Quantitative imaging features (radiomics) were extracted for both flanks. Subsequently, the treated animals received radiation (only to the right flank) in three 8 Gy fractions followed by PD-1 inhibitor administrations. Tumor volumes were followed and animals exhibiting identical of better tumor growth delay on the non-irradiated (left) flank as compared to the irradiated flank were identified as experiencing an abscopal effect. Binary logistic regression analysis was performed to create models for CT and MRI radiomics and blood counts, which are predictive of the abscopal effect.

**Results:**

Four of the treated animals experienced an abscopal effect. Three CT and two MRI radiomics features together with the pre-treatment neutrophil-to-lymphocyte (NLR) ratio correlated with the abscopal effect. Predictive models were created by combining the radiomics with NLR. ROC analyses indicated that the CT model had AUC of 0.846, while the MRI model had AUC of 0.946.

**Conclusions:**

The combination of CT and MRI radiomics with blood counts resulted in models with AUCs of 1 on the modeling dataset. Application of the models to the validation dataset exhibited AUCs above 0.84, indicating very good predictive power of the combination between quantitative imaging and blood counts.

## Introduction

Immunotherapy (IT) has revolutionized treatment and improved prognosis for patients with various cancers, including lung cancer [7]. In advanced non-small cell lung cancer (NSCLC), immune-checkpoint inhibitors targeting programmed cell death protein-1 (PD-1) can provide lasting treatment response and improve long-term survival [8,9]. Despite these successes, overall response rates to IT remain less than 50% and non-responding patients can experience accelerated disease progression [1–4]. Isolation of factors that predict patient response, such as programmed death ligand 1 (PD-L1) expression and tumor mutational burden, influence clinical decision-making regarding patient management selection.

IT and radiotherapy (RT) can act synergistically, enhancing antitumor response beyond what either treatment can achieve separately [13,15]. Anecdotal reports suggest that this results, in part, through induction of a systemic abscopal effect on non-irradiated lesions [5]. Systematic data on incidence of the abscopal effect and in particular on signatures or biomarkers predictive of the abscopal effect are lacking [5]. Thereby, it remains elusive which patients are most likely to achieve and benefit from this phenomenon. Literature review over the past 50 years [5] discovered 13 preclinical papers which have reported on the abscopal effect. According to the authors “*eleven preclinical papers used a combination of immune modification and radiotherapy to achieve abscopal effects*”, indicating that if abscopal effect is to be studied then a combined treatment including RT and IT needs to be utilized. These findings were furthermore substantiated by more recent work [6], where abscopal response was only observed in combined RT and IT treatments. Therefore, the pre-clinical work presented herein was performed with two murine cohorts: a control cohort receiving no treatment, and a treatment cohort where widely used combined RT and IT regimen [6] was adopted.

In this work it was hypothesized that the abscopal effect after the combined RT and IT regimen is a true phenomenon and can be predicted by pre-treatment CT and MRI radiomics [7–10], as well as by complete blood counts (CBCs). The novel findings related to the potential of quantitative imaging and CBCs to differentiate among animals which will experience the abscopal effect are presented below.

## Materials and methods

Since this is a proof-of-principle investigation there was no any *a priori* basis for sample size estimation. In a seminal work [6] of combined radioimmunotherapy it was observed that 2 out of 7 treated animals experienced an abscopal response. Therefore, in order to critically minimize the animal number usage, we designed the experiment was designed with 4 control and 18 treated (combined RT and IT) animals with the expectation that 4–5 of the treated animals would exhibit abscopal effect, which would allow the hypothesis in this proof-of-principle investigation, namely that the abscopal effect can be predicted by pre-treatment CT and MRI radiomics and CBCs. The study design and approach were approved by the Institutional Animal Care and Use Committee.

The approach undertaken for minimizing pain and distress in the laboratory animals was based on the following of the principles of the Three Rs—refinement, reduction, and replacement. The animals were maintained at the appropriate day-light cycle (12 hours light, 12 hours dark) to provide good suitable living conditions. All mice used in this study showed normal weight and physical activity when assessed on daily basis. Cleanliness of the cages was observed on daily, and all necessities were provided for animal good health and well-being. Food and water usage as well as physical movements were examined on routinely to ascertain normal physical and behavioral activity and good health. Five mice per cage were paired up to maximize the living space and reduce any in-bound fighting. Single housing was not encouraged in order to avoid any distress on the animal mental and physical health. Behavioral indicators including wounds and limping, hunched posture, dull or sluggish movements, large or open tumors, or no animal motion when the cage is manipulated were monitored on daily basis. Pain and distress were often assessed to establish that the animals were in good health by overall status. Physical examinations on face and mouth, feet and limb, genital abnormalities, and abdominal palpation were regularly performed. Skin lesions, lumps, eyes and surrounding tissues, and mobility issues were observed pre and post tumor implantation. Neurologic conditions including ataxia, head tilt, and head spinning were also monitored. Signs of respiratory distress include dyspnea, shallow rapid breathing, gasping, or abdominal effort in breathing were followed. Other general conditions for examining diarrhea, ascites, anasarca, rectal prolapse and rectal prolapse were monitored in order to seek medical help when needed. The supportive animal care that was provided through this study included morning and evening check-ups on the animals. Warming up measure were provided to the animals after anesthesia utilized for treatment or imaging. Furthermore, saline (as body fluid), soft-food pallets, and water gels were supplied to the animals after anesthesia to maintain their normal functioning.

The criteria used to determine if premature eutanisation was needed were based on clinical signs and observed physical and behavioral indicators such as rapid or progressive weight loss, debilitating diarrhea, dehydration/reduced skin turgor, edema, sizable abdominal enlargement or ascites, progressive dermatitis rough hair coat, hunched posture lethargy or persistent recumbency coughing, labored breathing, nasal discharge, jaundice, cyanosis, and/or pallor/anemia. Furthermore, any condition interfering with daily activities (e.g., eating or drinking, ambulation, or elimination) as well as excessive or prolonged hyperthermia or hypothermia was used in the eutanisation decision making. Based on those euthanisation criteria three animals were terminated since they experienced high rate of tumor growth within short span of time, thereby reducing the pain and the distress in the animals.

Lewis Lung Carcinoma (LLC) cells were implanted subcutaneously on both flanks in nineteen C57BL/6 mice. The cell line was cultured in medium Dulbecco’s Modified Eagle’s Medium (Gibco®; Thermo Fisher Scientific, Inc., Waltham, MA, USA), supplemented with 10% fetal bovine serum (Gibco®; Thermo Fisher Scientific Inc.) and 1% penicillin-streptomycin (HyClone; GE Healthcare Life Sciences, Logan, UT, USA). For subcutaneous implantation, a cell suspension of a density of 1 × 10^5^ cells/ml was prepared for each animal.

The mice (6–8 weeks old; 18–20 g) were purchased from Jackson Laboratory (Bar Harbor, ME, USA) and were housed at 22 ± 5°C in a 12 h light/dark cycle and fed rodent chow and water freely. Mice were subcutaneously inoculated with 100 μL LLC cell suspension (1 × 10^5^ cells) under 2% isoflurane. The skin was tented up, and the tumor cells were implanted under the skin in the dorsal regions of both left and right flanks. The tumor volume was measured twice or thrice a week by direct measurement with calipers and calculated using the formula, [width^2^ (mm^2^) × length (mm)]/2 (volume of an ellipsoid).

**Fig 1:**
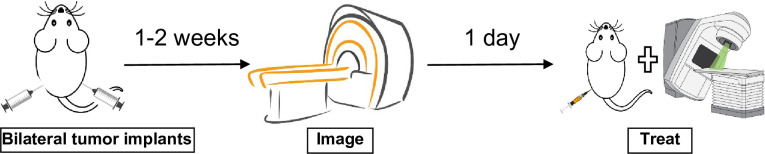
**Study design: From left to right–bilateral tumors are implanted and 1–2 weeks later (tumor volumes ~200 mm3) all animals are CT and MR imaged, radioimmunotherapy is started on the day after imaging, and is performed for three consecutive days.** On each treatment day tumors are irradiated with 8 Gy followed by PD-1 is administered through intraperitoneal injection. Final tumor volumes are measured 2 days post therapy completion.

**Fig 2:**
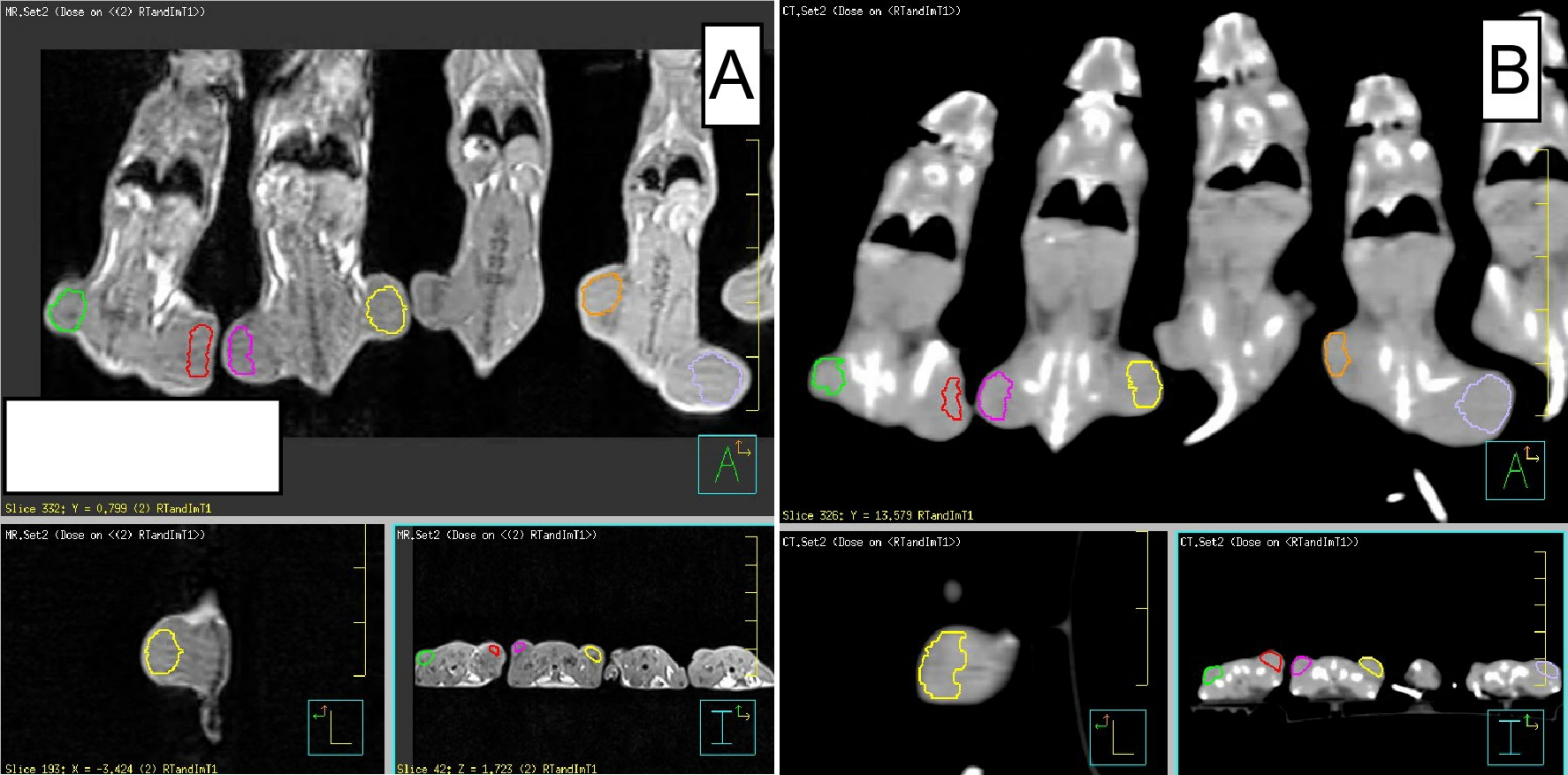
T1 3T MRI image (panel A) and CT image (panel B) of subcutaneos syngeneic lung mice tumors, implanted on flanks. The tumors are segmented for radiomics features extraction. The panels contain coronal (top), sagital (lower left), and axial (lower right) views.

Four mice were used for control, and fifteen were treated with a combined RT and IT regimen [6]. Approximately ten days after tumor implantation, when the tumors reached ~214 mm^3^ on average (range from 99.8 mm^3^ to 437 mm^3^), non-contrast MRI- and CT-imaging were performed. **Fig 1** outlines the experimental design according to which the animals were inoculated, imaged, and treated. The tumor growth variability is in accordance with Giraldi et al [11] and the doubling time is approximately a week. For CT scans, a resolution of 0.4×0.4×0.6 mm^3^ was used, while for the MR imaging a T1 sequence with image resolution of 0.5×0.5×0.5 mm^3^ was employed. The CT hardware was 64-slice Siemens (Erlangen, Germany) Somatom Definition AS scanner, while the MRI hardware was 3T Siemens (Erlangen, Germany) TrioTim scanner. After imaging the tumors on both flanks were segmented as outlined on **Fig 2**where CT (left) and MRI (right) screen captures are shown.

Quantitative imaging features (radiomics) were then extracted from the imaging studies [8–10,12,13]. The radiomics were computed with in-house developed software [7,14]. The MRI radiomics were extracted after intensity normalization [15–17], which is a pre-processing step deemed appropriate for minimizing inter-subject variance due to MRI scanner parameters.

In addition to the CT and the MRI imaging features, blood was collected on the day of imaging and CBCs were performed. Approximately 100–200 μl of blood were collected from each mouse by retro-orbital method and was suspended in an eppendorf tube pre-loaded with 10 μl of 0.5M EDTA. Subsequently the whole blood was mixed properly by pipetting up and down. The flowing analysis of CBCs was processed by using automated blood counter HESK (Version Element H5). The data was collected from the blood counter and was assessed to look at each component of the CBCs. The derived CBCs components included white blood cells, neutrophils, lymphocytes, monocytes, eosinophils, basophils, red blood cells, etc. Furthermore, ratios of those CBCs were also used in the downstream categorization.

After imaging, the animals were irradiated using a RadSource 2000 X-Ray Irradiator cabinet and organ-specific irradiation jigs (160 kVp, 25 mA, 0.5 mM Cu, 1.8 Gy min-1) under 2% isoflurane. The RT involved daily treatment for three consecutive days to the **right** flank only with daily doses of 8 Gy [6]. On each of these 3 days, RT was immediately followed by intraperitoneal injections of 200μg of PD-1mAb (BioXcell anti-mouse PD-1 (CD279)). Local tumor control was evaluated by measuring tumor volumes with digital calipers. The measurements were performed for the tumors on both flanks, thereby tracking the irradiated (primary) site and the non-irradiated (abscopal) site simultaneously. The animals exhibiting abscopal effect were identified based on the tumor volume evolution of the non-irradiated tumors (left flank) as cross-compared to the tumor volume evolution of the irradiated ones (right flank).

92 CT and 92 MRI radiomics features for each tumor were extracted [2]. For each imaging modality the features were divided into four groups–geometric features, first order histogram features, second-order joint probability features (e.g. co-occurrence matrices), and third-order joint probability features, originally described by our group (more details are in the supplementary material) [2]. The geometric, first-, and second-order features [10] are among the most commonly used features in the radiomics studies, while the third-order joint probability features have been developed by our group [7]. Radiomics studies use a wide range of features which they report on—from couple dozens to couple thousands. The studies with fewer radiomics usually use simpler features which are easier to interpret, while the studies with large number of features involve more convoluted ones, thereby being more difficult to understand. The choice of the quantitative imaging features presented herein was based on their widespread use and their somewhat easer interpretation. All of the radiomics utilized in this study were extracted with in-house developed software [7,14,18] interfaced with the Pinnacle (Philips Radiation Oncology Systems, Madison, WI) treatment planning system.

The treated animals were divided into two groups–one for predictive model generation consisting of 6 animals, and another one for model validation consisting of 9 animals. Since the imaging features were extracted for both right- and left-flank tumors before treatment, form modeling perspective there is no difference whether the imaging features come from the right flank (treated with RT) or from the left flank (abscopal). In other words, the imaging/CBCs predictive of abscopal effect should be pertinent for both tumors regardless of which tumor was treated with RT and which one was used as an abscopal site, since the choice of treatment site is purely arbitrary. After feature extraction, ANOVA (SPSS Statistics V.25 software package, IBM Corp., Armonk, NY, USA) analyses was performed among all features and the dichotomized (yes/no) abscopal effect as well as all the CBCs and the abscopal effect in the modeling cohort. The imaging features and CBCs with highest statistical significance were selected. Furthermore, all selected imaging features and CBCs were tested for correlation, where Pearson correlation coefficient of 0.5 was used for cut-off. After inter-correlated features were removed, the remaining uncorrelated features and CBCs were subjected to binary logistic regression, aiming to model the abscopal effect for the training cohort. The results of the converged combined (imaging + CBCs) models were applied to the validation cohort and the results were tallied.

## Results

**Fig 3:**
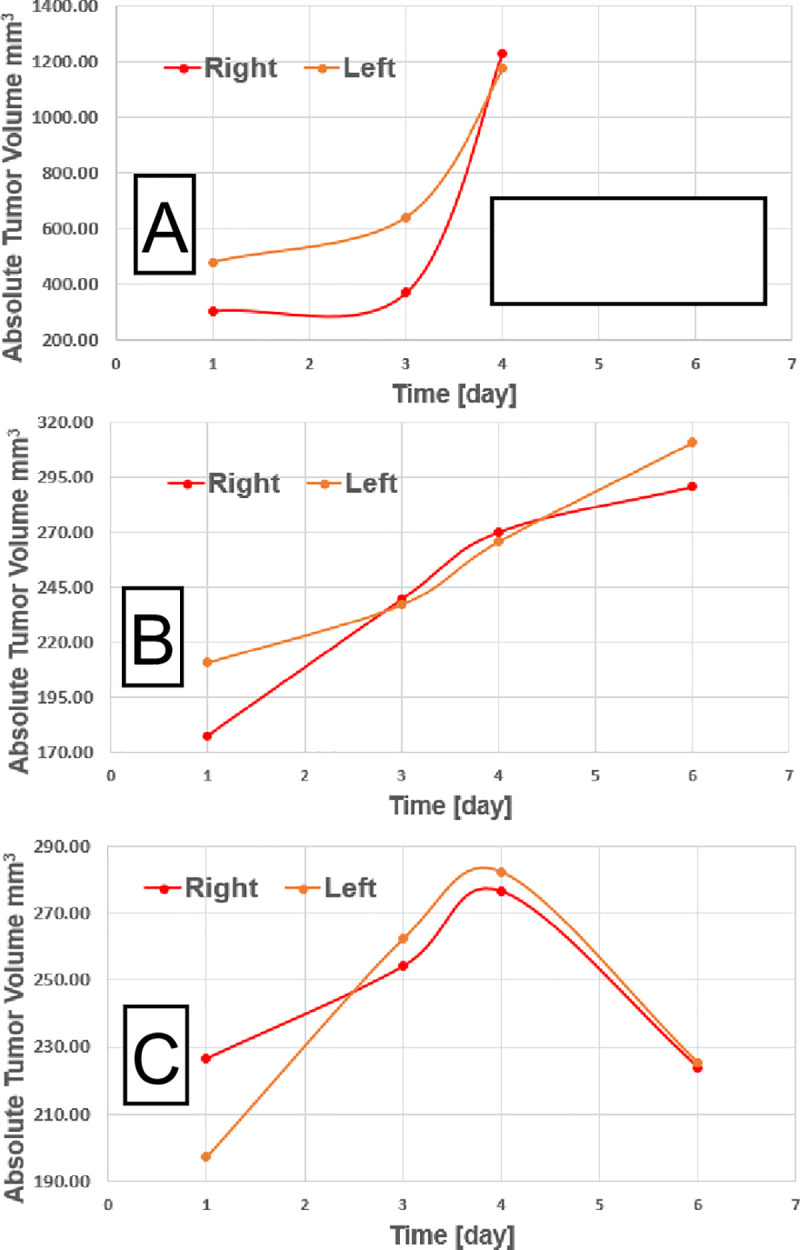
Tumor size temporal evolution for several animals. The red line depicts right-flank (treated with RT) tumor volume, while the orange line is left-flank tumor volume. The volumes are absolute and day 1 is at time of CT- and MRI-imaging. A) Control animal (untreated) tumors, with rapid growth, B) Delayed tumor growth, but no abscopal since the untreated with RT tumor (left) grows faster than the treated with RT tumor (right), C) Abscopal effect case, since both treated with RT (right) and untreated with RT (left) tumors respond similarly with time.

Approximately 27% (4 out of 15) of the treated animals experienced an abscopal effect. **Fig 3** demonstrates the tumor growth evolution, used to determine the abscopal response. The abscopal effect was defined either as identical growth delay in both right- and left-flank tumors, or as augmented growth delay in the left-flank (*non-irradiated*) tumor. Notably, all tumors in the treated cohort experienced growth delay and thereby demonstrated response to therapy as compared to the control (untreated) cohort. With that definition of abscopal effect in mind the top panel (panel **A**) in **Fig 3**outlines the tumor growth in an untreated (control) animal. The control animals were sacrificed before the final tumor volume measurements since the tumor growth continued beyond IACUC (protocol: 17-214-ad02 EDR) limits. Panel **B** of the figure demonstrates no abscopal effect, since the left-flank tumor (not treated with RT) grows faster than the right-flank (treated with RT) tumor. Finally, panel **C** demonstrates the *abscopal* effect, where the left- and the right-flank tumors exhibit same temporal evolution after combined radioimmunotherapy treatment, where they shrink simultaneously.

Based on the abscopal effect presented on **Fig 3**, 3 CT radiomics features were selected for correlation with the abscopal response–Surface-To-Mass Ratio, 2D (Histogram) Kurtosis, and Average Gray Value. For the MRI radiomics the selected features which correlated with the abscopal effect were 2D (Histogram) Kurtosis and 3D information measure of correlation (IMC1) [2]. The CBCs which was also indicative of the abscopal response was the pre-treatment neutrophil-to-lymphocyte ratio (NLR).

For the CT features the surface-to-mass ratio is not dimensionless, and therefore it is (partly) dependent on the mass of the tumor. Here, a lower value indicates a more compact tumor. The surface-to-mass ratio for the tumors in the animals experiencing abscopal effect is larger indicating less compact tumors. The histogram from which the 2D kurtosis was derived was constructed by using 128 equally-spaced bins covering the range of gray values in the image. Kurtosis is the fourth central moment of a distribution, and it measures the ‘peakedness’ of the distribution of values in the histogram. A higher kurtosis implies that the mass of the distribution is concentrated towards the tail(s) rather than towards the mean. A lower kurtosis implies the reverse: that the mass of the distribution is concentrated towards a spike near the mean value. The 2D kurtosis for the tumors in the abscopal cohort is smaller than the tumors in the non-abscopal cohort indicating broader distribution of the histogram. Finally, the average gray values for the tumors in the abscopal group is smaller than average gray values for the tumors in the animals which did not exhibit abscopal effect, indicating less dense tumors.

The 2D kurtosis in the MRI images was derived similarly to the 2D kurtosis in the CT images–the normalized gray values in the tumors were binned into 128 equally-spaced bins covering the entire gray value range. The kurtosis for the tumors in the abscopal group is approximately four times larger than the kurtosis in the non-abscopal tumors, indicated much more “peaked” distribution in the MRI histogram. MRI 3D IMC1 assesses the correlation between the probability distributions of *i*, *j* and *k* tumor voxels (see supplementary material for definition) using mutual information, and therefore quantifying the complexity of the texture. In the case where the distributions are independent, there is no mutual information, and the result will therefore be 0. The average 3D IMC1 for the abscopal tumors is smaller than the average 3D IMC1 for the non-abscopal tumors. Finally, the average NLR for the abscopal cohort was approximately 66% of the NLR for the animals which did not experience an abscopal effect.

**Fig 4:**
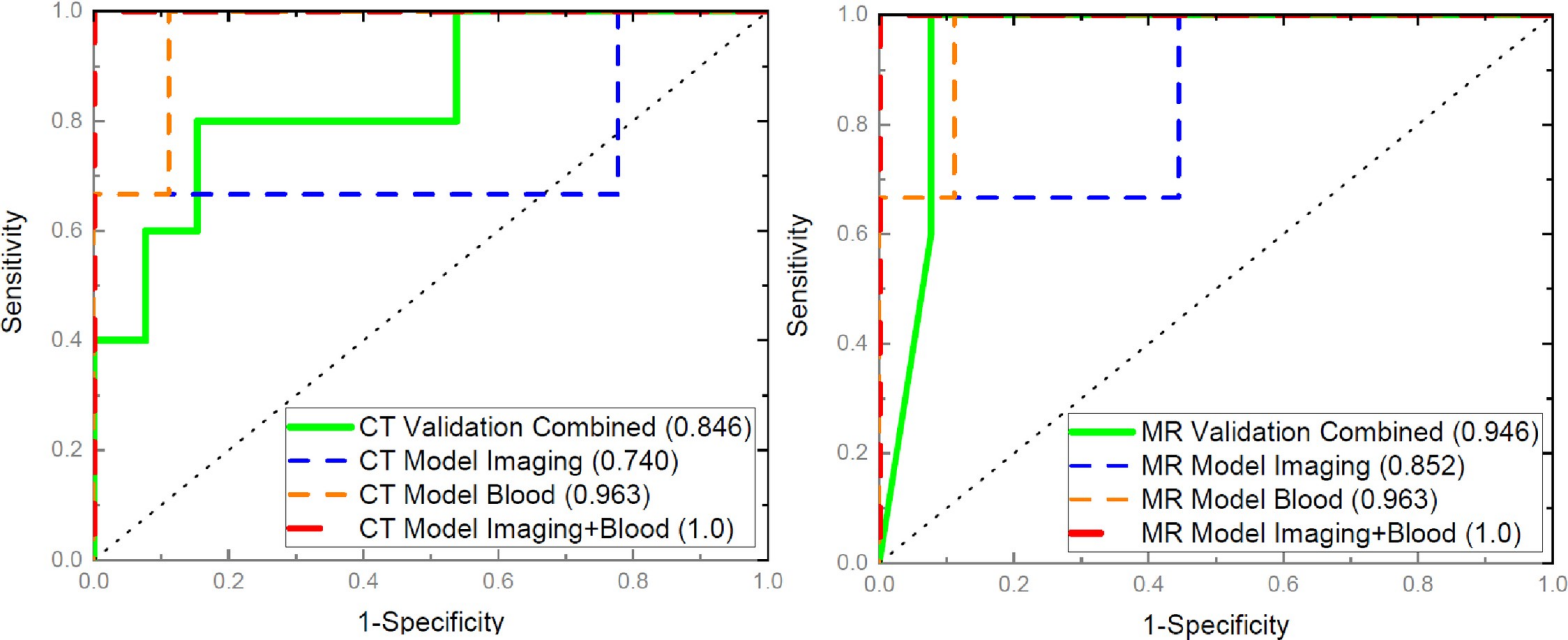
CT (left) and MRI (right) ROC curves for selected radiomics features, blood counts, and composite radiomics and blood counts, which exhibit statistical significance in predicting abscopal effect as inferred form **Fig 2**. The dashed lines are from the training set, where the blue lines are from radiomics (CT: surface-to-mass ratio +2D Kurtosis+Average Gray; MRI: 2D Kurtosis+3D IMC), the orange lines are from blood counts (neutrophil-to- lymphocyte ratio), and the red lines are from the combined radiomics and blood counts. The solid lines are from the vlaidation set, where the green lines are rthe cruves from the combined binary logistic model including radiomics and blood counts. The corresponding AUCs are presented in parethesis in each figure legend.

The results for the binary logistic regression of pretreatment imaging features, pretreatment CBCs, and combination of both to the abscopal effect are quantified on **Fig 4**, where several AUCs are outlined. The dashed lines are the AUCs from the model training (**S1 Appendix**) cohort of 6 animals (40% of the entire cohort) chosen randomly, or total of 12 tumors. The AUCs for imaging features (blue), NLR (orange), and combined imaging and NLR (red) are presented. The left panel is for the CT studies where the imaging features of interest are Surface-to-Mass ratio, 2D Kurtosis, and Average Gray, while the right panel depicts the MRI results where the imaging features of interest are 2D Kurtosis and 3D IMC1. The corresponding AUC for the CT imaging features is 0.74 (95% CI: 0.44 to 1.03), for the NLR is 0.96 (95% CI: 0.82 to 1.09), and for the combination is 1 (95% CI: 1.0 to 1.0). The AUC for the MRI imaging features is 0.85 (95% CI: 0.6 to 1.1) and for the combined MRI radiomics and NLR is 1.0 (95% CI: 1.0 to 1.0). The solid lines (green) in both panels outline the AUCs when the CT and the MRI models (corresponding imaging features combined with NLR) are applied to the validation (**S1 Appendix**) cohort of 9 animals, or 18 tumors. The CT AUC is 0.846 (95% CI: 0.65 to 1.05) while the MRI AUC is 0.946 (95% CI: 0.77 to 1.17).

These findings indicate that the abscopal effect after RT (24 Gy total dose, delivered in three equal-sized fractions) followed by IT is a true phenomenon and can be modeled by pretreatment CT and MRI radiomics combined with pretreatment blood counts.

## Discussion and conclusions

Heterogeneity in patient response to tumor therapies complicates clinical decision-making and patient selection for treatment. A pivotal advance in cancer care in the last decade is undoubtedly IT which, in combination with existing therapies, can dramatically improve patient outcomes. Sources of heterogeneity include tumor and patient genetics, prior exposure to varied therapies, and stochastic differences in tumor microenvironments. In the present study, we assessed the predictive power of pre-treatment radiomics and complete blood counts on the abscopal effect in a defined syngeneic murine model of lung cancer. By using murine tumors derived from the same cell line, we removed genetic variation, yet still observed significant variations in response. Therefore, differences in tumor growth and in the abscopal response due to therapy can be attributed to the microenvironment of the tumor in the host animal. These animal-specific microenvironments resulted in distinct tumor phenotypes which correlated well with abscopal response and were captured by both CT and MRI quantitative imaging. In addition, the tumor microenvironment affected blood counts in the different animals such that pretreatment neutrophil-to-lymphocyte ratio was also indicative of abscopal response. The blood count correlations are not surprising since an elevated neutrophil count can stimulate tumor angiogenesis and contribute to disease progression or resistance to therapy. Therefore, fewer neutrophils and more lymphocytes in the pretreatment state would seem to correlate with better treatment response (lower NLR) as indicated by several studies [19,20]. According to a published clinical study [21] "*high pretreatment neutrophil-to-lymphocyte ratios and lymphocytopenia were associated with poor outcomes*" and the reverse is true for lower NLR. The authors point out that circulating T-cell counts dropped an average of nearly 2-fold at the end of SBRT (1074 cells/μL to 556 cells/μL) and subsequently returned to near baseline levels after 3 months. This was due to peripheral T-cells "*trafficking to the lymph nodes and the tumor microenvironment*, *both at the irradiated site and potentially to metastatic sites*”. Therefore, a high absolute peripheral lymphocyte count and a low NLR pre-treatment means that there is a potential for good radioimmunotherapy response.

To our knowledge this is the first preclinical study where the abscopal effect has been studied in a methodical fashion for treatment naïve animals in the context of quantitative imaging and blood counts. Despite the relatively small cohort size of only fifteen animals, it was observed that the abscopal effect is experienced in more than one quarter of the animals, when a combined RT and IT treatment regimen was employed. The obtained area-under-the-curve measurements for both imaging modalities were all larger than ~0.84, indicating very good to excellent predictive power with radiomics combined with lab data. It should be noted however that caution must be exercised in not overinterpreting or overgeneralizing the findings presented here in. This proof-of-principle investigation was performed in a single syngeneic subcutaneous murine tumor model and needs to be generalized in other tumor models.

Future directions of the research will include studies where the model will be extended to other tumor types, which will improve the significance of the identified features and CBCs. In addition, longer follow up times will be utilized in an effort to capture more detailed and representative tumor evolution patterns. Molecular biomarker extraction from tissue and blood samples will add another dimension toward the understanding of the interaction of different predictive features. Additionally, the molecular biomarkers will allow more mechanistic framework for prediction of this complex phenomenon called abscopal effect.

## Supporting information

S1 Appendix Model data. (XLSX)
